# Innervation of Gill Lateral Cells in the Bivalve Mollusc *Crassostrea virginica* Affects Cellular Membrane Potential and Cilia Activity

**Published:** 2016-03-26

**Authors:** Edward J Catapane, Michael Nelson, Trevon Adams, Margaret A Carroll

**Affiliations:** Department of Biology, Medgar Evers College, 1638 Bedford Ave, Brooklyn, NY, 11225, USA

**Keywords:** *Crassostrea virginica*, DiBAC_4_(3), Membrane potential, Cilia

## Abstract

Gill lateral cells of *Crassostrea virginica* are innervated by the branchial nerve, which contains serotonergic and dopaminergic fibers that regulate cilia beating rate. Terminal release of serotonin or dopamine results in an increase or decrease, respectively, of cilia beating rate in lateral gill cells. In this study we used the voltage sensitive fluorescent probe DiBAC_4_(3) to quantify changes in gill lateral cell membrane potential in response to electrical stimulation of the branchial nerve or to applications of serotonin and dopamine, and correlate these changes to cilia beating rates. Application of serotonin to gill lateral cells caused prolonged membrane depolarization, similar to plateau potentials, while increasing cilia beating rate. Application of dopamine hyperpolarized the resting membrane while decreasing cilia beating rate. Low frequency (5 Hz) electrical stimulations of the branchial nerve, which cause terminal release of endogenous serotonin, or high frequency (20 Hz) stimulations, which cause terminal release of endogenous dopamine, had the same effects on gill lateral cell membrane potentials and cilia beating rate as the respective applications of serotonin or dopamine. The study shows that innervation of gill lateral cells by the branchial nerve affects membrane potential as well as cilia beating rate, and demonstrates a strong correlation between changes in membrane potential and regulation of cilia beating rate. The study furthers the understanding of serotonin and dopamine signaling in the innervation and regulation of gill cilia in bivalves. The study also shows that voltage sensitive fluorescent probes like DiBAC _4_(3) can be successfully used as an alternative to microelectrodes to measure changes in membrane potential of ciliated gill cells and other small cells with fast moving cilia.

## Introduction

**Figure 1:**
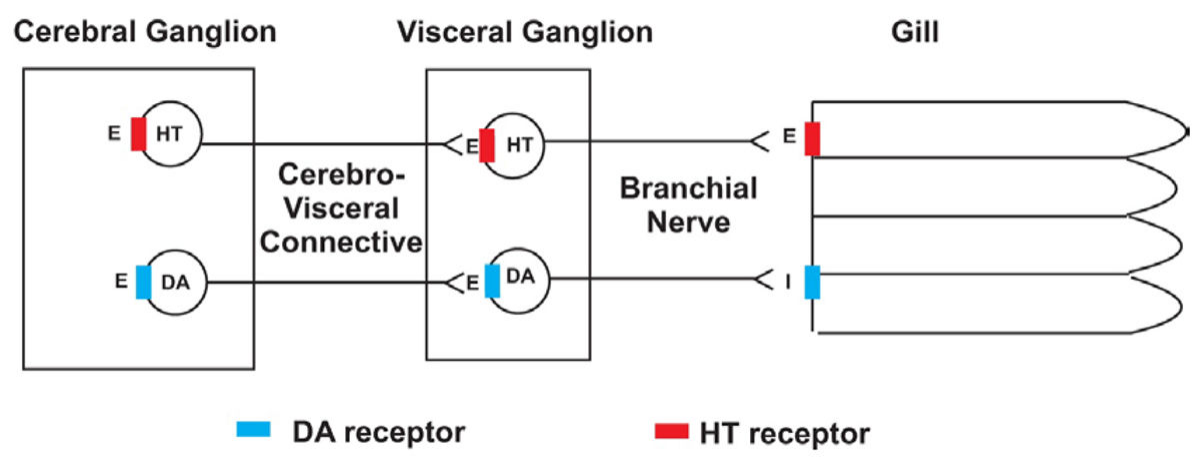
Schematic of the innervation of gill lateral cell cilia by the cerebral and visceral ganglia via the cerebrovisceral connective and the branchial nerve (from Carroll and Catapane [4]).

Gills of bivalve molluscs function in both respiratory and feeding roles. Cilia are present on gill filaments in lateral, latero-frontal and frontal cells and each type has specific arrangements and functions. Frontal and latero-frontal cell cilia are involved with moving particles along the gill surface, while the lateral cell cilia generate the water currents that provide for gas exchange as well as food intake and waste removal [1]. In both *Mytilus edulis* and *Crassostrea virginica* a wealth of histological, pharmacological, neurochemical and physiological studies have shown that ciliary activity in the lateral cells is regulated by serotonin and dopamine, which function as both peripheral and ganglionic neurotransmitters. Serotonergic and dopaminergic nerves are present in the cerebrovisceral connective, which connects the cerebral and visceral ganglia, as well as in the branchial nerve that emerges from the visceral ganglia to innervate the gill [2,3]. Figure 1 is a schematic showing this innervation in *C. virginica* [4] and is representative of other bivalves with innervated lateral gill cells. The lateral cell cilia beat in a metachronal wave fashion allowing their beating rate to be measured by stroboscopic light [5]. Gill lateral cell cilia beating rate is increased in a dose dependent manner by super fusion of serotonin to gill cells or ganglia; in contrast super fusion of dopamine to gill cells or ganglia decreases cilia beating rate [2–4,6–12]. Using suction electrodes to stimulate the cerebrovisceral connective or branchial nerve results in terminal release of these neurotransmitters at the gill. Low frequency (5 Hz, 2 ms duration) stimulation releases endogenous serotonin, which increases the beating rate of lateral cell cilia; while high frequency stimulations (20 Hz, 2 ms duration) releases endogenous dopamine, which decreases the beating rate [11–13].

Recently our lab identified a dopamine D2-like, metabotropic receptor responsible for slowing cilia beating rates in gill lateral cells of *C. virginica* by using a combination of pharmacological and immunohistofluorescence techniques with dopamine receptor agonists, antagonists and antibodies [14]. Since activation of dopamine D2 receptors has been shown to hyperpolarize innervated cells, we hypothesized that the decrease in cilia beating rate caused by dopamine would correlate with a hyperpolarization of gill lateral cells. Likewise, since serotonin increases cilia beating rate, we hypothesize that serotonin would depolarize the gill cells. Since using microelectrodes to measure membrane potentials in these small ciliated oyster gill cells can be problematic, we employed a voltage-sensitive probe to optically quantify changes in membrane potential in gill lateral cells. DiBAC_4_(3) (Bis-(1,3-dibutylbarbituric acid) trimethine oxonol) is a slow-response, voltage-sensitive probe that has been used to optically quantify changes in membrane potential [15,16]. DiBAC_4_(3) enters depolarized cells where it binds to intracellular proteins or membrane, but is excluded from mitochondria because of their overall negative charge, making this voltage-sensitive probe superior to carbocyanines for measuring plasma membrane potentials. Membrane depolarization causes DiBAC_4_(3) to exhibit a red spectral shift and an increase in green fluorescence, while hyperpolarization decreases green fluorescence [17]. Unlike using microelectrodes which may damage these small ciliated cells, this method will allow us to optically measuring changes in membrane potentials while stroboscopically measuring cilia beating rates of the same cells in response to electrical stimulation of the branchial nerve, or to bath application of serotonin and dopamine.

## Materials and Methods

### Chemicals

Dopamine HCl and serotonin creatinine sulfate monohydrate were obtained from Sigma-Aldrich. Just prior to use, serotonin was dissolved in artificial sea water (ASW, Instant Ocean Aquarium Systems) and dopamine was dissolved in ASW containing 10 mg% ascorbic acid (ASWA) buffered with sodium bicarbonate (pH 7.0) to retard dopamine oxidation [18]. The fluorescent probe DiBAC_4_(3) was obtained from AnaSpec, Inc., and stock and working solutions were stored in brown bottles. DiBAC_4_(3) stock solutions (24 mM) were dissolved in DMSO and working solutions (2.4 μM) were freshly prepared in ASW. All other chemicals were obtained from Fisher Scientific.

### Oyster maintenance and gill preparations

Adult oysters of approximately 80 mm shell length were obtained from Frank M Flower and Sons Oyster Farm in Oyster Bay, NY, USA. Oysters were maintained for up to two weeks in temperature regulated aquaria in ASW at 16–18°C, specific gravity of 1.024 “ 0.001, 31.9 ppt salinity and pH of 7.8 “ 0.2. Gill preparations consisted of a small section of oyster gill (4 mm wide) with the branchial nerve, which continues through the gill axis, attached. Each preparation was placed on a microscope slide with a cover slip. The cover slip was supported by thin spacers that prevented crushing of the filaments and allowed for fluid movement between the slide and cover slip.

### Cilia activity and membrane potential observations

**Figure 2:**
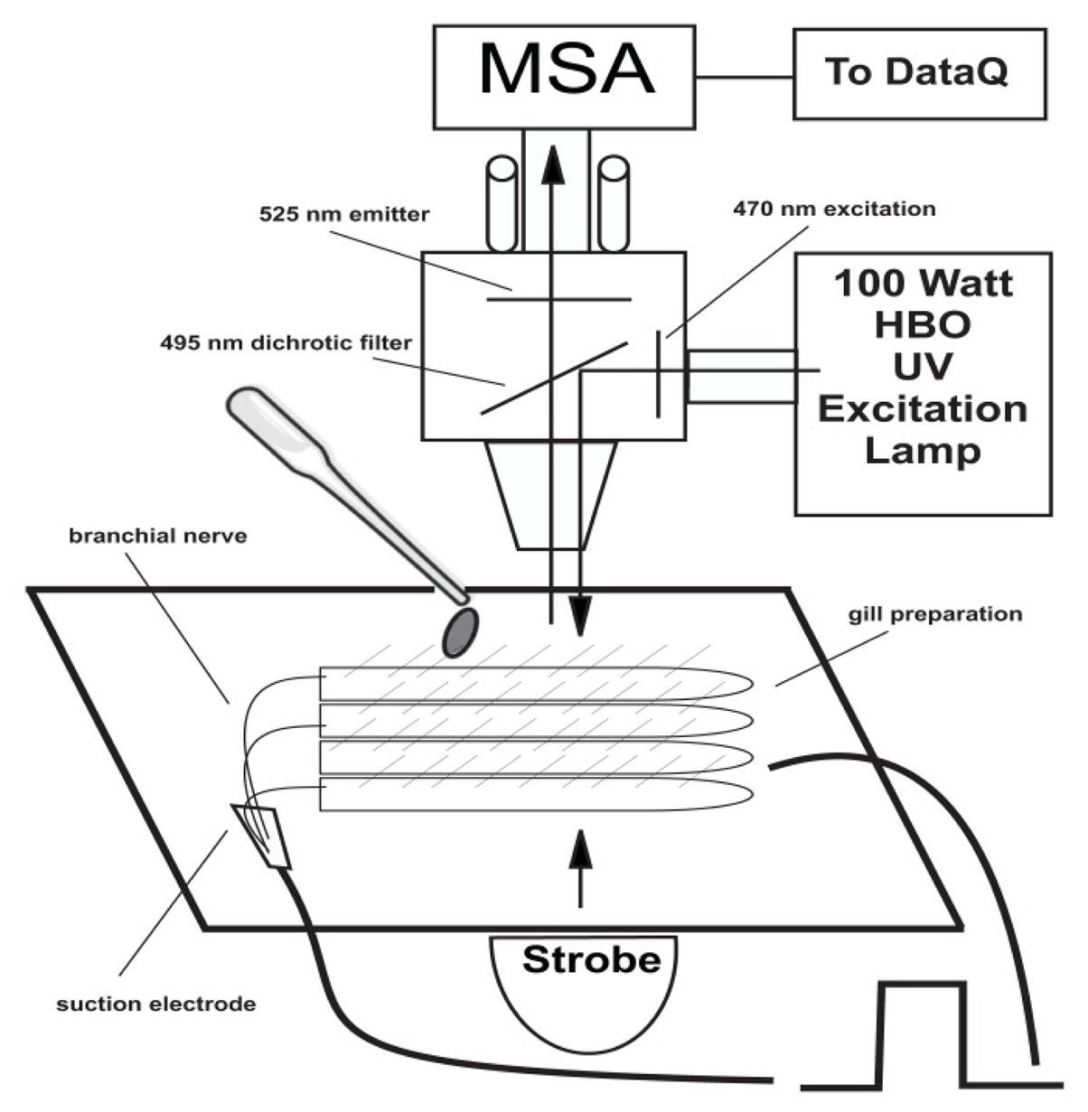
Schematic of Zeiss epilume fluorescence microscope fitted a Farrand Microspectrum Analyzer (MSA) for measuring fluorescence intensities. A stroboscope provided transmitted light for measuring cilia beating rate. Electrical stimulations were applied to the branchial nerve in the gill axis using suction electrodes and drugs were applied to the medium bathing the gill.

Gill preparations were tested for innervation integrity by test stimulation to the branchial nerve and observing the expected change in cilia beating rate. Biphasic electrical stimulations to the branchial nerve (5 or 20 Hz, 2 ms duration, 10 V) were applied using a Grass SD9 Stimulator. Suction electrodes were made from polyethylene tubing sized to fit the diameter of the nerve. Gill lateral cell cilia beating rates were measured by synchronizing the flashing rate of the stroboscope with the beating rate of the cilia [10]. To determine changes in membrane potentials working solutions of DiBAC_4_(3) were added to gill preparations for 10 min for baseline readings, followed by either drug superfusion or branchial nerve stimulations. Gill preparations were viewed at 200× magnification with a Zeiss epilume fluorescence microscope. The microscope was fitted with transmitted stroboscopic light from a Grass PS 33 Plus Photic Stimulator as well as incident light illumination from a 100 W HBO mercury vapor lamp and an ET-GFP-FITC/Cy2 filter set from Chroma Technologies (470 nm excitation, 525 nm emitter and 495 nm dichroic filters). A Farrand Instruments Mircospectrum Analyzer was attached to the microscope and had a 0.55 mm diameter ocular target window, which with 200× magnification, enabled viewing and recording of both fluorescence intensities (μA) at 516 nm and cilia beating rates (beats/s) from individual gill lateral cells (Figure 2). For each DiBAC_4_(3) treated gill preparation, lateral cells along a gill filament were individually positioned in the target window so that cilia beating rates and fluorescent intensities could be recorded before and after various treatments. For each experiment the cilia beating rates of individual lateral gill cells along a gill filament were stroboscopically determined and then fluorescence intensity of the same cells was measured by switching the filters to the incident light source. Fluorescence data were collected and analyzed with a DI-700 Data Acquisition System from DATAQ Instruments. Representative photomicrographs of gill filaments were taken with a ProgRes C3 camera.

### Statistical analysis

Cilia beating rates (beats/s) and fluorescence intensity are expresses as mean ± sem. Statistical analysis was determined by one-way ANOVA with Tukey Post Test.

## Results

### Fluorescence microscopy

**Figure 3:**
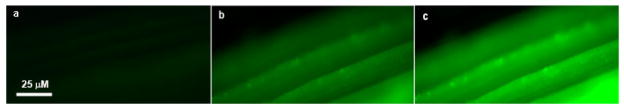
Photomicrographs of the effects of 10 μM serotonin (HT) on DiBAC_4_(3) fluorescence intensity of the same gill filaments. (a) before application of DiBAC_4_(3), (b) after application of DiBAC_4_(3) and (c) after application of serotonin. Sections were viewed with 470 nm excitation, 525 nm emitter and 495 nm dichroic filters, and photographed with a ProgRes C3 with the same exposure settings.

**Figure 4:**
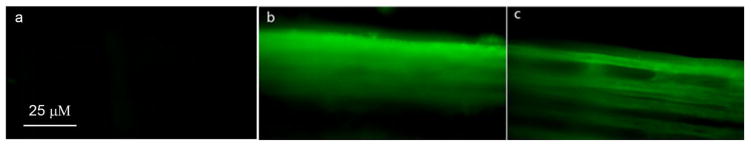
Photomicrographs of the effects of 10 μM dopamine (DA) on DiBAC_4_(3) fluorescence intensity (a) before application of DiBAC _4_(3), (b) after application of DiBAC_4_(3) and (c) and after application of dopamine. Sections were viewed with 470 nm excitation, 525 nm emitter and 495 nm dichroic filters, and photographed with a ProgRes C3 with the same exposure settings.

Figures 3 and 4 are representative photomicrographs of gill preparations illuminated by the HBO mercury vapor lamp and viewed using the ET-GFP-FITC/Cy2 filter set demonstrating the development of the green fluorescence due to DiBAC_4_(3). Figure 3a is before DiBAC_4_(3) application showing a lack of auto or background fluorescence, while Figure 3b is the same gill filaments 10 min after DiBAC_4_(3) application. DiBAC_4_(3) caused the filaments to display a dim green fluorescence in the lateral and other gill cells, indicating a negative resting membrane potential. Figure 3c illustrates the effects of 10 μM serotonin (ED_50_ concentration [4]) on the membrane potential and shows a dramatic increase in green fluorescence intensity indicating gill cell membrane depolarization. Figure 4 is a series of similar photomicrographs of a new gill preparation showing an opposite effect on membrane potential upon dopamine application. Figure 4a is before DiBAC_4_(3), Figure 4b is 10 min after DiBAC_4_(3), and Figure 4c shows a decrease in green concentration [4]) indicating fluorescence after 10 μM dopamine (ED_50_ hyperpolarization of the gill cell membrane.

### Response of cilia and membrane potential to serotonin and dopamine

**Figure 5:**
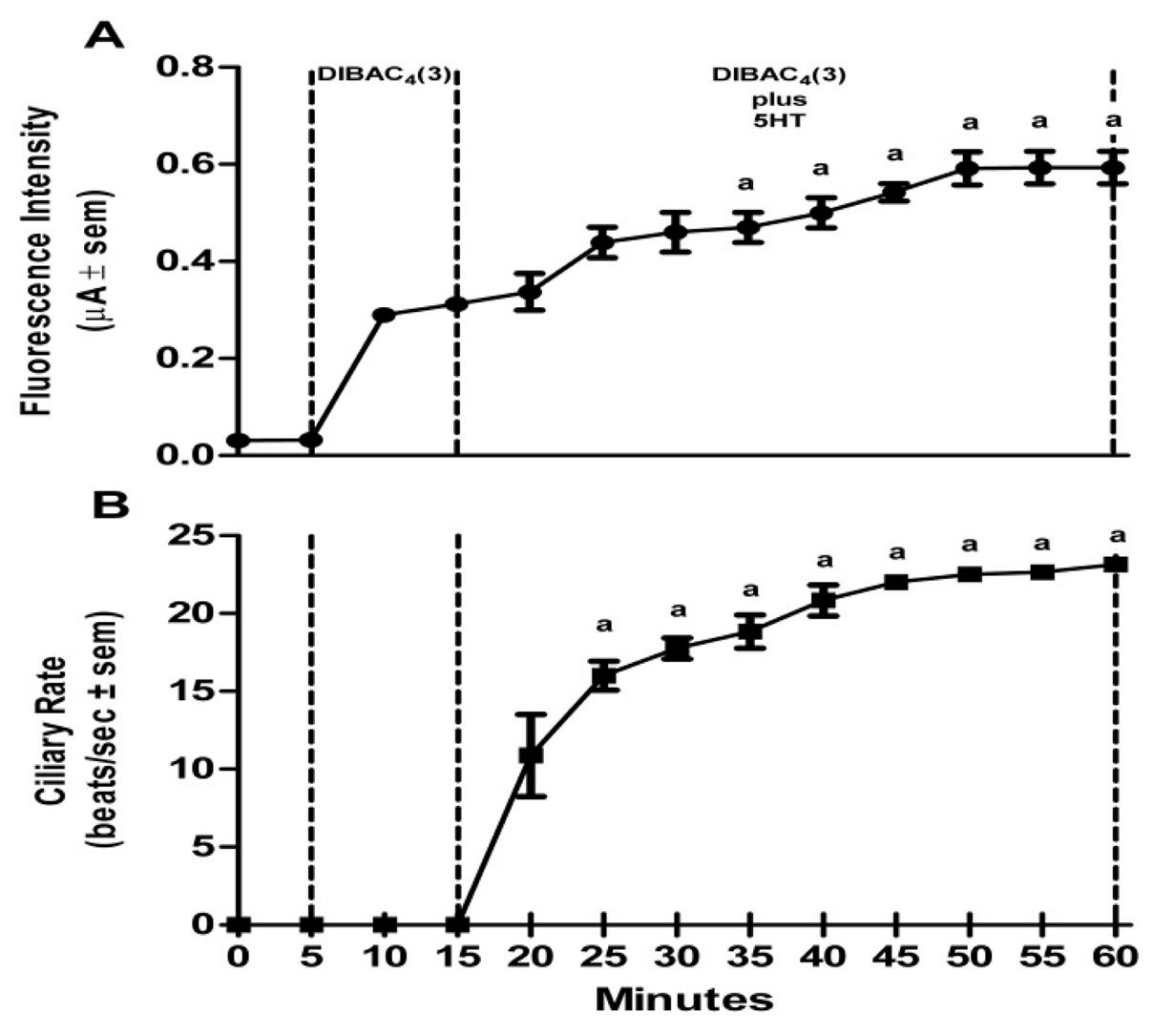
Effects of HT on changes in DiBAC_4_(3) fluorescence intensity and cilia beating rate in response to application of 10 μM serotonin (HT) to gill filaments over a 60 min period. (A) change in fluorescence intensity (μA ± sem), (B) the change in beating rate of lateral cell cilia (beats/s ± sem). Statistical analysis was determined by a one way ANOVA with Tukey post-test, ^a^ p<0.001 compared to the reading after 10 min of DiBAC_4_(3), number of preparations (n) =8.

**Figure 6:**
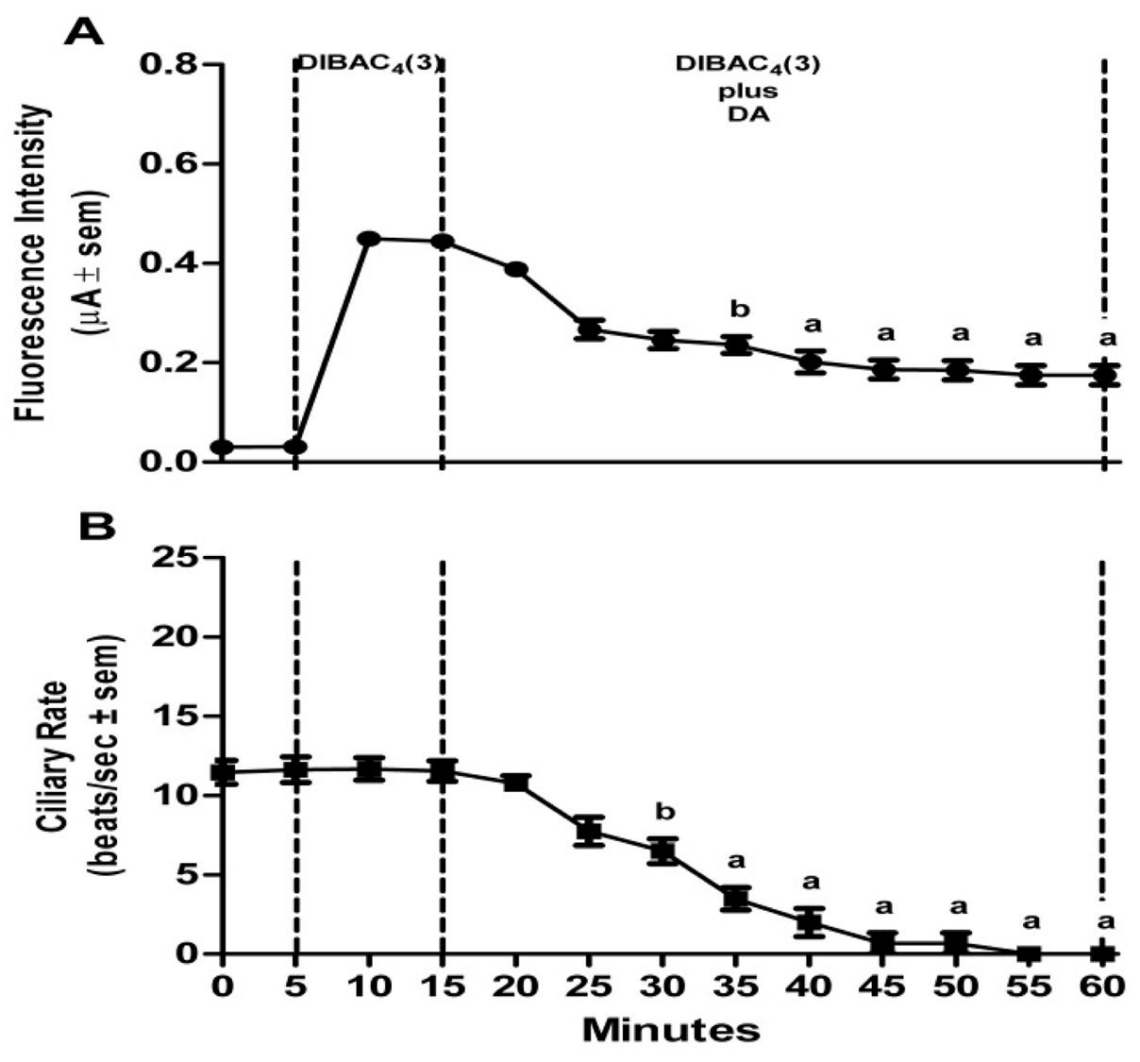
Effects of DA on changes in DiBAC_4_(3) fluorescence intensity and cilia beating rate in response to application of 10 μM dopamine (DA) to gill filaments over a 60 min period (A) change in fluorescence intensity (μA ± sem), (B) the change in beating rate of lateral cell cilia (beats/s ± sem). Statistical analysis was determined by a one way ANOVA with Tukey post-test, ^a^p<0.001, ^b^p<0.01 compared to the reading after 10 min of DiBAC_4_(3), number of preparations (n) =8.

Lateral cells of isolated, unstimulated, gill preparations typically had cilia beating rates of zero, while some preparations start off with basal beating rate as high as 10–15 beats/s. In experiments to test stimulatory effects, gill preparations with low basal lateral cell beating rate (under 5 beats/s) were selected, whereas in experiments to test inhibitory effects, gill preparations with high basal cilia beating rates were selected. Applying DiBAC_4_(3) (2.4 μM) to gill preparations had no effect on the beating rate of the lateral cell cilia. The changes in DiBAC_4_(3) fluorescence intensity and cilia beating rate in response to serotonin or dopamine applications are shown in Figures 5 and 6, respectively. Addition of serotonin (10 μM) resulted in an increase in DiBAC_4_(3) fluorescence intensity and was positively correlated with an increase in the beating rate of the lateral cell cilia (Figure 5). In other preparations, the addition of dopamine (10 μM) caused the opposite effects, decreasing both DiBAC_4_(3) fluorescence intensity and cilia beating rate (Figure 6). The effects of serotonin or dopamine application on both fluorescence intensity and cilia beating rate were long lasting, at least for 45 min.

### Response of cilia and membrane potential to electrical stimulations

**Figure 7:**
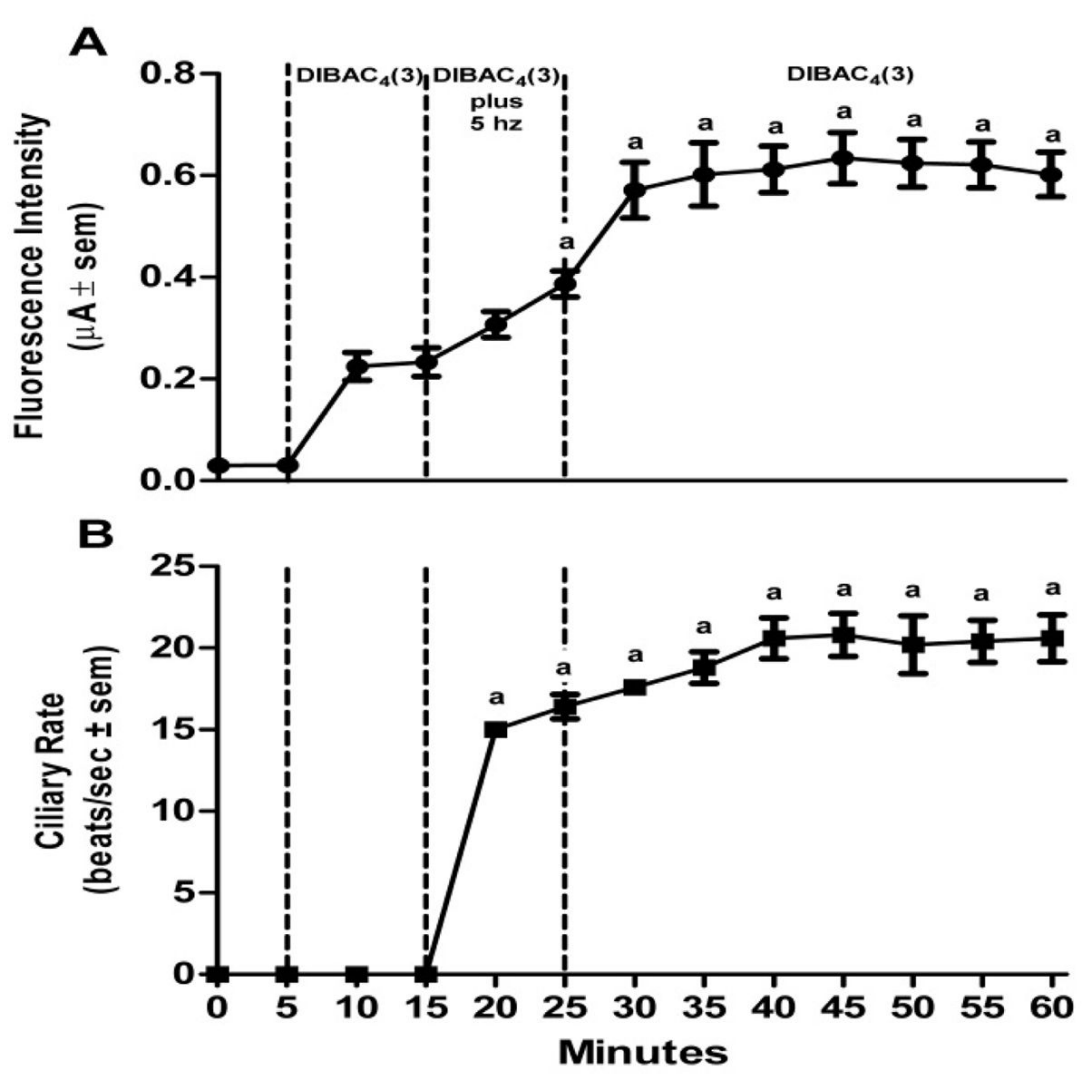
Effects of low frequency electrical stimulation to the branchial nerve on DiBAC_4_(3) fluorescence and cilia beating rate of gill filaments over a 60 min period. (A) change in fluorescence intensity (μA ± sem), (B) the change in beating rate of lateral cell cilia (beats/s ± sem). The branchial nerve was stimulated at 10 V, 2 ms duration and 5 Hz using suction electrodes. Statistical analysis was determined by a one way ANOVA with Tukey post-test, ^a^p<0.001, ^b^p<0.01 compared to readings after 10 min of DiBAC_4_(3), number of preparations (n) =10.

**Figure 8:**
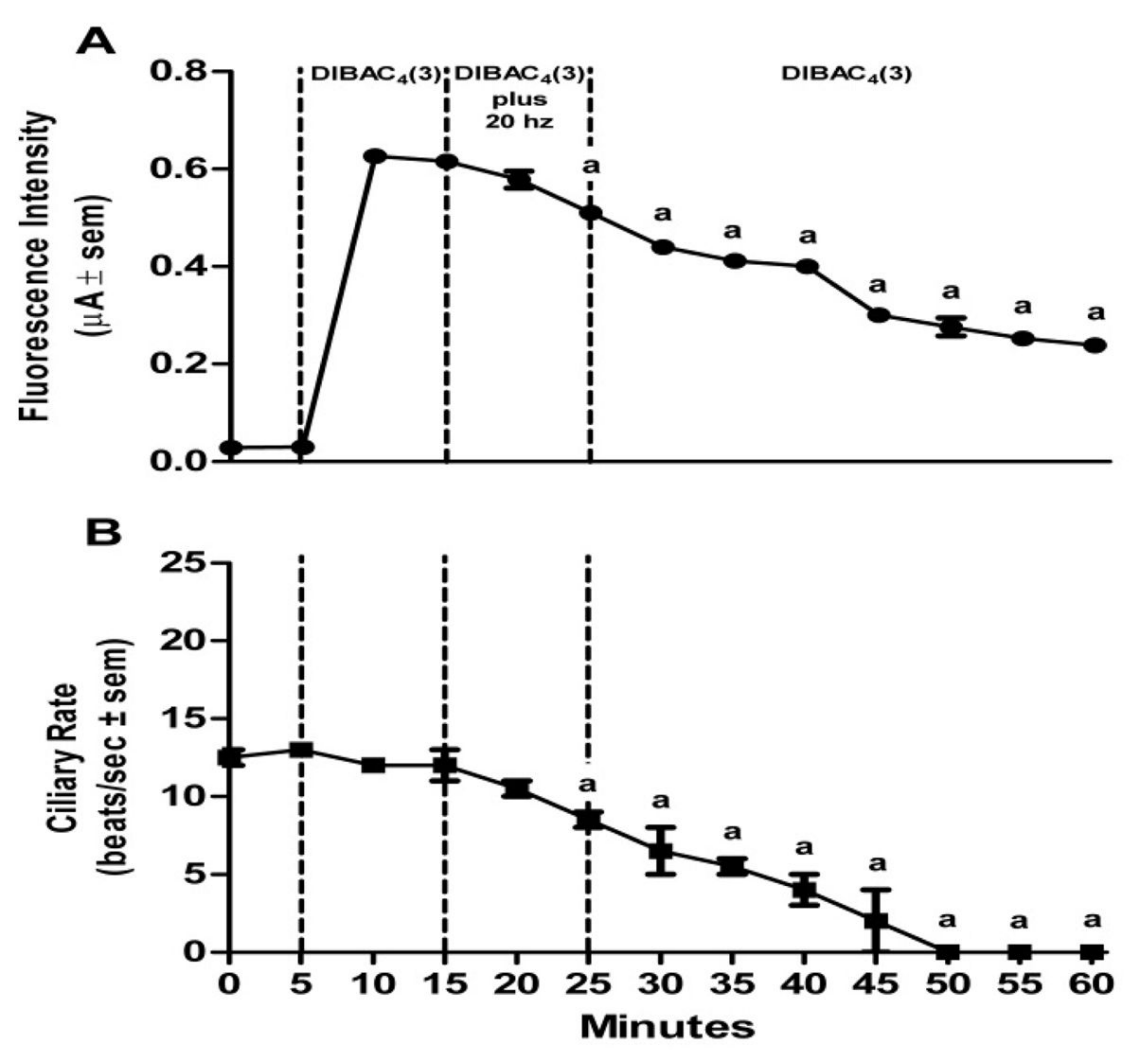
Effects of high frequency electrical stimulation to the branchial nerve on DiBAC_4_(3) fluorescence and cilia beating rate of gill filaments over a 60 min period. (A) change in fluorescence intensity (μA ± sem), (B) the change in beating rate of lateral cell cilia (beats/s ± sem). The branchial nerve was stimulated at 10 V, 2 ms duration and 20 Hz using suction electrodes. Statistical analysis was determined by a one way ANOVA with Tukey post-test, ^a^ p<0.001 compared to readings after 10 min of DiBAC_4_(3), number of preparations (n) =10.

Since we previously showed in *C. virginica* that electrical stimulations to the branchial nerve elicit a terminal release of endogenous serotonin or dopamine causing the respective ciliary response in the lateral cells, we conducted experiments to determine if electrical stimulations caused similar changes in DiBAC_4_(3) fluorescence intensity as did exogenous application of serotonin and dopamine. Using suction electrodes the branchial nerve was stimulated at either 5 or 20 Hz and changes in both DiBAC_4_(3) fluorescence intensity and cilia beating rate of lateral cells were measured. Repetitive biphasic stimulations over a 10 min period at low frequency (5 Hz, 2 ms duration, 10 V) steadily increased both DiBAC_4_(3) fluorescence intensities and cilia beating rate (Figure 7) whereas repetitive high frequency stimulation (20 Hz) had the opposite effect, decreasing DiBAC_4_(3) fluorescence intensities and cilia beating rate (Figure 8). These effects also were long lasting with changes in fluorescence intensities and cilia beating rate continuing for at least 30 min after stopping stimulations.

## Discussion

Changes in membrane potential can be measured optically using a variety of slow response molecular probes. In this study we were able to use the voltage-sensitive fluorescent probe DiBAC_4_(3) to correlate changes in fluorescent emission with changes in cilia beating rate in lateral cells of gill of *C. virginica.*

Historically, using intracellular microelectrodes to measure membrane potential of ciliated cells has mainly been carried out on large protozoans and has demonstrated that ciliary activity in these cells is associated with electrical properties of the cell membrane [19,20]. However, inserting microelectrodes into small ciliated cells such as those found in the gill of bivalves is problematic due to the fast moving cilia breaking microelectrode tips and tearing open the cells [21,22]. While control of ciliary activity in metazoans, including bivalves, has been well studied by pharmacological and physiological methods, reports involving the recording of membrane potentials in small ciliated cells have rarely been reported beyond the electrophysiological studies of Murakami and Takahashi [23] and Saimi et al. [24] Working with *M. edulis*, they were able to elegantly use microelectrodes with tips fashioned to reach around the cilia to insert into gill lateral cells to record membrane potentials in experiments lasting for seconds. In gill cells that were not damaged Murakami and Takahashi [23] determined that lateral ciliated cells have a resting membrane potential of about −60 mV. They further found that stimulating the branchial nerve with a single 2 ms pulse caused a transient ciliary arrest and depolarization of about 20 mV. Using the same technique, Saimi et al. [24] correlated the increase cilia beating rate of gill lateral cells due to serotonin application to membrane depolarization. The more recent development of voltage sensitive molecular probes like DiBAC_4_(3) enables longer term experiments and overcomes the difficulties of using microelectrodes in the small ciliated gill cells of *C. virginica*. While this method does not directly measure the absolute voltage of the membrane potential, it does allow one to optically quantify changes in DiBAC_4_(3) fluorescence intensity due to changes in membrane potential.

The present study shows that innervation of the gill lateral cells by the branchial nerve affects membrane potential as well as cilia beating rate, and that there is a strong correlation between changes in membrane potential and regulation of cilia beating rate. Membrane depolarization due to serotonin super fusion or endogenous serotonin release after low frequency electrical stimulations correlated with increased lateral cell cilia beating rate. Membrane hyperpolarization due to dopamine super fusion or endogenous dopamine release after high frequency electrical stimulations correlated with decreased cilia beating rate.

Assuming the gill lateral cells of *C. virginica* have a typical resting membrane potential of −60 mV, similar to that reported in *M. edulis* [24], our results from the experiments reported in Figure 7 indicate that 10 min of excitatory electrical stimulations (5 Hz) increased fluorescence intensity by about 60%, which would correspond to a depolarization of the membrane by approximately 36 mV, and 10 min of inhibitory stimulation (20 Hz) decreased fluorescence intensity by about 20%, which would correspond to a hyperpolarization of the membrane by approximately 12 mV. These numbers are in line with the findings of Murakami and Takahashi [23] and Saimi et al. [24], when they made direct measurements using microelectrodes on *M. edulis* gill. They also fit well with the DiBAC_4_(3) approximation of a 1% change in fluorescence intensity equaling about a 1 mV potential change. The membrane potential changes as well as the changes in cilia beating rate were long lasting and extended beyond the cessation of stimulations, indicative of plateau potential responses. The increased cilia beating rate could last up to at least an hour after low frequency stimulation. When the cilia were slowed with high frequency stimulation, the cilia would tend to continue to beat slowly until reactivated with serotonin super fusion, application of excitatory electrical stimulations, or washed several times.

The relationships between membrane potential and cilia beating has been studied in cultured cells and a variety of ciliates, invertebrates and vertebrates, with different results depending on the organisms. In general, it is fairly well established that a rise in intracellular Ca^2+^ enhances ciliary motility in organisms that have been studied [25–29]. Studies with tissue cultures grown from excised frog (*Rana ridibunda*) palate and esophagus show that increased cytosolic Ca^2+^ induces the opening of calcium-activated K^+^ channels causing membrane hyperpolarization and an increase of the ciliary beat frequency [30]. In paramecium and other large ciliates, changes in beat frequency and beat orientation are correlated with changes in membrane potential [31–34]. Saimi and Kung [35] and Bonini et al. [36] found that both depolarization and hyperpolarization tended to increase cilia beating rate, but depolarization often produced cilia reversals and backwards swimming motions. In the gastropod *Calliostoma ligatum,* the beating frequency of the locomotory, pre-oral cilia of competent veligers is dependent upon the level of membrane depolarization, and is modulated by excitatory neuronal input, which slows down the inherent beating frequency [37].

## Conclusion

The study provides a foundation with which to further investigate the pharmacological and toxicological relationships between membrane potential and physiological responses of ciliated cells of bivalve gill. The study also demonstrates that voltage sensitive probes such as DiBAC_4_(3) can be successfully used to determine these relationships in small ciliated cells of organisms that have proved difficult to study with microelectrodes.

## Abbreviations

DiBAC_4_(3): Bis-(1,3-Dibutylbarbituric Acid) Trimethine oxonol
Hz: Cycles per Second (Hertz)
ms: Milliseconds
ASW: Artificial Sea Water
ASWA: ASW with Ascorbic Acid
DMSO: Dimethyl Sulfoxide
HT: Serotonin
DA: Dopamine
μM: Micromoles/Liter
mm: Millimeter
ppt: Parts per Thousand
nm: Nanometer
μA: Microamperes
s: Seconds
ED_50_: Median Effective Dose
mV: Millivolts
